# Therapeutics of the Throat

**Published:** 1888-03

**Authors:** 


### THERAPEUTICS OF THE THROAT.

### ARUM TRIPHYLLUM.

*Objective. Lividity.*

Mucus: tenacious: with tickling, compelling cough.

*Return of drink through nose on swallowing.*

Swelling: right side: both sides: of region of larynx, causing cough: of tonsils: extending toward nose.

*Ulceration: of fauces and nares, with acrid secretion: deep and angry-looking, with fetid discharge.*

*Subjective.* BURNING: *with pain all day: at root of tongue: with dryness* before midnight: in morning while in bed: with soreness and pain in palate, worse eating or drinking:

*with scratching and desire to swallow: with stinging:* with rawness: of pharynx and glottis: as of something hot, worse during inspiration: *in morning while in bed, better after rising:* OF TONGUE: WITH SORENESS AND PAINS.

*Constriction: as if too narrow:* with sneezing.

*Dryness:* before midnight.

*Mucus (sensation of): after midnight, goes lower and lower by swallowing, and is perceived no more after rising in morning:* through posterior nares, with soreness.

*Pain:* worse at night: worse coughing: with burning: *on pressure:* in palate, with soreness and burning, worse eating and drinking: in larynx, constant.

RAWNESS (sensation of): AT ROOT OF TONGUE; OF PALATE: with burning, *scratching:* better after drinking hot coffee: *with burning, with desire to swallow.*

*Soreness: at 4 p. m.:* with cough at 5 A. M.: with burning and pain in palate, worse eating or drinking: with sensation of much mucus through the posterior nares: WITH BURNING: OF TONGUE WITH BURNING: *going from right to left.*

Stinging with burning.

*Stitches:* left side: *with constant desire to swallow.*

*Swallowing difficult:* in morning, but without pain.

*Swelling* (sensation of): *in soft palate, or swallowing in morning.*

*Aggravation or cause.*

Afternoon 4 o'clock (soreness).

Breakfast, better after.

Chewing, while.

Coughing (pain).

Dinner, after.

Drinking, while (burning, soreness, and pain in palate).

Eating, " " " " " hoarseness.

Inspiring, while (burning, as from something hot).

Midnight, before (dryness, burning).

" after (sensation of mucus there).

Morning, (soreness and burning of mouth, lips, and soft palate—hawking of mucus): sensation of swelling. Discharge of mucus from nose. MOUTH, LIPS, AND PALATE SORE. Hawking of mucus.

Morning, on swallowing.

" 5 o'clock (soreness and cough).

Morning, while in bed (burning).
Night (pain).
Overuse of voice (hoarseness).
Pressure (pain).
Swallowing (sprained feeling in left maxillary articulation).
" in morning.
Talking (hoarseness).

*Amelioration.*

Drinking hot coffee (scratching).
Breakfast, after.
Morning, till after breakfast.
" after rising (burning).
Rising (mucus).
Talking a little (hoarseness).

*Concomitants.*

Bleeding and rawness of buccal cavity.
Boring into nose with fingers.
BURNING OF MOUTH, LIPS, AND SOFT PALATE, WITH SORENESS, IN MORNING: OF MOUTH, SO THAT THEY REFUSE DRINK, AND CRY WHEN IT IS OFFERED.
CHAPPED FEELING OF NOSE, LIPS, AND FACE.
*Chilly over whole body, beginning in vertex.*
*Cold, sensation as if he had taken.*
*Coryza fluent and acrid.*
CRACKED AND SORE CORNERS OF MOUTH.
Delirum at times.
DIPHTHERITIC DEPOSITS COVERING CAVITY OF MOUTH.
Dryness of nose and *mouth.*
Hawking in morning.
HOARSENESS worse before talking, better after moderate use of voice; worse from overuse of voice in speaking or singing; worse by taking food.
Irritable.
*Mucus, from nose, with streaks of blood and hardened pieces in morning: yellow and black from nose during day:* CONTINUALLY FROM LEFT NOSTRIL: TOUGH, FROM MOUTH: *Acrid and ichorous from nose, excoriating inside of alæ and upper lip: yellow and corrosive, from nose in diphtheria.*
OBSTRUCTION OF NOSE, COMPELLING TO BREATHE THROUGH MOUTH: odor from mouth putrid. Œdema glottidis. *Left side of nose: of nose, with watery discharge.*
*Pain in left maxillary articulation, as if sprained on swallowing.*

PICKING LIPS TILL THEY BLEED : *at ends of fingers: at nose : at one spot.*
*Rawness of nostrils :* WITH BLEEDING OF BUCCAL CAVITY.
Restlessness.
*Salivation, excessive and acrid.*
Screaming.
*Sneezing.*
Soreness, OF NOSTRILS : OF CORNERS OF MOUTH : of lips and soft palate : *of mouth, so that he was unwilling to drink,* AND CRIES WHEN IT IS OFFERED, WITH BURNING : *of left parotid : of mouth, causing sleeplessness.*
*Stiffness of neck.*
Swelling OF GLANDS OF NECK : OF LEFT SUB-MAXILLARY GLAND : OF BOTH SUBMAXILLARIES.
ULCERS IN CAVITY OF MOUTH.
Uncertain, uncontrollable voice.
*Urine scant or suppressed.*
Wakefulness.

*Remarks.*

Aphonia after exposure to northwest wind, or when singing.
APHTHOUS SORE THROAT.
Clergyman's sore throat.
DIPHTHERIA, with putrid odor.
Putrid sore throat.
*Scarlatina.*
Typhoid condition.
Compare Am. mur., Castor., Cep., K. iod., Lyc., Mez., Nit. ac., Sil. (nasal discharge).
Arg. m., Crocus (tongue).
Caps. (throat).
Caust., Fer. phos. (hoarseness).
Merc., Verat. (hips and corners of mouth).

WM. JEFFERSON GUERNSEY, M. D.

---

### BAPTISIA TINCTORIA.

OBJECTIVE.—Fauces dark red ; dark, putrid ulcers. · Tonsils and parotids swollen (*unusual absence of pain*). Putrid sore throat. Ulcers in the mouth. Tonsils and soft palate swollen with constant inclination to swallow, not accompanied by pain. Tonsils and soft palate very red, but not painful. Tonsils and soft palate congested. Diphtheria with dark membrane in the throat.

SUBJECTIVE.—Pain and soreness of the fauces. Hot feeling in the fauces passing up into ears; sensation as if had eaten pepper. Scraping and burning; raw sensation in the pharynx, much viscid mucus; copious flow of saliva; dry scraping in the throat when swallowing. Stitches in the right tonsils; pricking in the upper part of pharynx; raw sensation. Tickling in the throat provoking cough; uvula elongated. Sore throat; averse to open air. Mucus abundant and viscid, can neither be swallowed nor expectorated. Mucus rattles in the throat. Constricted feeling causing frequent efforts at deglutition. Throat sore, feels contracted. Throat feels swollen, full (frequent inclination to swallow, causing pain at the root of the tongue). Spits out liquids put into the mouth. Can swallow liquids only, the least solid food gags. Children cannot swallow solid food; the smallest solid substance causes gagging; thus they cannot use anything but milk. Unable to swallow. Paralysis of organs of deglutition. Sore throat extending to the posterior nares; the throat feels sore and contracted. Diphtheria with fœtid breath. Ulceration of the throat and great prostration.

CONCOMITANTS.—*Stupid and drowsy, can with difficulty be aroused; goes to sleep while answering a question; lies in a stupor; a besotted look.* Nose and cheeks very red. Fan-like motion of the alæ nasi. (Lyc., Phos., Brom., Ant. tart.) Aching all over from the end of the nose to the ends of the toes. Aching deep in the bones. Bed feels as hard as a board. (Arn., Rhus.) Great restlessness, but worse from motion, like Bry. alb. Vertigo on rising from recumbent position, (Bry. alb., Phyto.) Numbness of the face, head, and hands. Numbness of the lips and tongue. The tongue feels swollen. Numbness all over the body. The patient fears paralysis. The pillow feels as hard as a rock. Tongue coated white, later dark down the centre. *The sweat, the urine, the stools all have a fœtid odor.*

REMARKS.—This valuable remedy has been too often overlooked in diseases of the throat. It differs from all of the remedies in our materia medica. In this respect the *absence of pain on swallowing is very peculiar*, and ought not to be forgotten. It has the horrible fœtor often found in diphtheria and ulceration of the mouth and fauces. It has the aching in the back like Phyto., but in Baptisia the aching is deeper in the limbs, as if in the bones. Phyto. has extreme sensitiveness of the throat and tonsils, while Baptisia has the absence of pain. Baptisia has cured ulceration of the mouth, with stringy, ropy saliva, clear and transparent; fully two quarts secreted in the twenty-four hours; so tenacious as to hang in ropes from the mouth to the floor; in this respect resembling (Kali bich.). The odor in such cases is terrible and nearly drives one from the room. These symptoms are found reliable in all diseases.

GEO. W. SHERBINO, ABILENE, TEXAS.

